# Supplementary material for: Clinical Benefit from Docetaxel +/− Ramucirumab Is Not Associated with Mutation Status in Metastatic Non-Small-Cell Lung Cancer Patients Who Progressed on Platinum Doublets and Immunotherapy
Source: Cancers (Basel). 2024 Feb 26;16(5):935. doi: 10.3390/cancers16050935 (PMC10931294; doi:10.3390/cancers16050935)
Supplement: Supplementary file 1 [file cancers-16-00935-s001.zip › Supplement files/Supplement1.pdf]

Supplementary Table S1. Baseline Numbers of Patients in Each Treatment Group

	**Overall (N=454)**	**MDA cohort (N=304)**	**Mayoclinic cohort (N=150)**
**Docetaxel monotherapy N=178**	N=178	N=99	N=79
**Docetaxel+Ramucirumab N=203**	N=203	N=142	N=61
**Platinum-Taxane-ICI-AI N=35**			
Carboplatin+Paclitaxel+Atezolizumab+Bevacizumab	N=35	N=28	N=7
**Platinum-Taxane-AI N=6**			
Carboplatin+Paclitaxel+Bevacizumab	N=6	N=4	N=2
**Platinum-Taxane-ICI N=19**			
Carboplatin+Paclitaxel+Atezolizumab	N=2	N=1	N=1
Carboplatin+Docetaxel+Pembrolizumab	N=4	N=4	N=0
Carboplatin+Paclitaxel+Pembrolizumab	N=10	N=10	N=0
Carboplatin+Paclitaxel+Nivolumab	N=1	N=1	N=0
Cisplatin+Paclitaxel+Pembrolizumab	N=2	N=2	N=0
**Platinum-Taxane N=13**			
Carboplatin+Docetaxel	N=7	N=7	N=0
Carboplatin+Paclitaxel	N=6	N=6	N=0

Abbreviations: ICI, Immune checkpoint inhibitors; AI, anti-angiogenesis inhibitor.

Supplementary Table S2. Mono-Variate Analysis for Selected Factors for PFS and OS of Patients with Docetaxel+/-Ramucirumab Treatment

Characteristics	Docetaxel Monotherapy				Docetaxel+Ramucirumab				Docetaxel+/-Ramucirumab			
	PFS		OS		PFS		OS		PFS		OS	
	HR(95% Cl)	P value	HR(95% Cl)	P value	HR(95% Cl)	P value	HR(95% Cl)	P value	HR(95% Cl)	P value	HR(95% Cl)	P value
**Age**		0.59		0.83		0.97		0.19		0.79		0.41
≥65	1		1		1		1		1		1	
＜65	1.09(0.80 -1.48)		1.04(0.75 -1.45)		0.99(0.74 -1.33)		0.80(0.57 -1.12)		1.03(0.83 -1.27)		0.90(0.71 -1.15)	
**Gender**		0.38		0.68		0.07		0.006*		0.045*		0.022*
Male	1		1		1		1		1		1	
Female	0.87(0.64 -1.18)		0.93(0.67 -1.30)		0.76(0.56 -1.02)		0.78(0.66 -0.89)		0.81(0.65 -1.00)		0.76(0.60 -0.96)	
**Smoking history**		0.71		0.09		0.58		0.95		0.91		0.25
Current/Former	1		1		1		1		1		1	
Never	0.94(0.66 -1.33)		0.72(0.49 -1.05)		1.10(0.79 -1.52)		1.01(0.83 -1.22)		1.01(0.80 -1.29)		0.85(0.65 -1.12)	
**Histology**		0.60		0.08		0.33		0.11		0.26		0.015*
ADC	1		1		1		1		1		1	
Non-ADC	1.11(0.74 -1.67)		1.46(0.96 -2.23)		1.22(0.81 -1.84)		0.83(0.65 -1.04)		1.18(0.89 -1.57)		1.47(1.08 -2.02)	
**ECOG**		0.08		0.004*		0.36		0.022*		0.05*		<0.001*
≥2	1		1		1		1		1		1	
0-1	0.73(0.52 -1.04)		0.57(0.39 -0.83)		0.85(0.60 -1.21)		0.63(0.4- 0.9)		0.78(0.61 -1.00)		0.59(0.45 -0.78)	
**Line of treatment**		0.60		0.60		0.62		0.37		0.84		0.60
≥3	1		1		1		1		1		1	
2	1.09(0.79 -1.49)		1.10(0.78 -1.55)		0.93(0.69 -1.25)		1.08(0.91 -1.28)		0.98(0.79 -1.21)		0.94(0.74 -1.19)	

**Brain metastasis**													
	Yes	1	0.16	1	0.42	1	0.85	1	0.11	1	0.50	1	0.44
	No	0.80(0.58 -1.09)		0.87(0.62 -1.22)		1.03(0.77 -1.38)		1.32(0.94 -1.86)		0.93(0.75 -1.15)		1.10(0.87 -1.40)	
**Liver metastasis**			0.90		0.14		0.11		0.75		0.19		0.62
	Yes	1		1		1		1		1		1	
	No	1.02(0.70 -1.50)		0.74(0.50 -1.11)		1.33(0.94 -1.88)		0.97(0.80 -1.18)		1.18(0.92 -1.52)		0.93(0.71 -1.23)	
**Bone metastasis**													
	Yes	1	0.50	1	0.84	1	0.73	1	0.26	1	0.46	1	0.59
	No	0.90(0.66 -1.22)		1.04(0.74 -1.45)		1.03(0.89 -1.19)		1.10(0.93 -1.32)		0.92(0.75 -1.14)		0.94(0.74 -1.19)	
**Adrenal metastasis**													
	Yes	1	0.022*	1	0.36	1	0.38	1	0.40	1	0.35	1	0.88
	No	0.64(0.44 0.94)		0.83(0.55 -1.24)		0.91(0.73 -1.13)		0.90(0.70 -1.15)		0.88(0.66 -1.16)		0.98(0.71 -1.34)	

Abbreviations: ICI, Immune checkpoint inhibitors; AI, anti-angiogenesis inhibitors; NSCLC, non-small cell lung cancer; TX, treatment; ECOG PS, Eastern Cooperative Oncology Group performance status; ADC, adenocarcinoma; SQC, squamous cell carcinoma; HR, hazard ratio.

Supplementary Table S3. Multi-variate Analysis for Selected Factors for PFS and OS of Patients with Docetaxel+/-Ramucirumab Treatment

Characteristics	Docetaxel Monotherapy				Docetaxel+ Ramucirumab				Docetaxel+/-Ramucirumab			
	PFS		OS		PFS		OS		PFS		OS	
	HR(95% Cl)	P value	HR(95% Cl)	P value	HR(95% Cl)	P value	HR(95% Cl)	P value	HR(95% Cl)	P value	HR(95% Cl)	P value
**Gender**					/	/						
Male							1	0.008*	1	0.041*	1	0.03*
Female							0.63(0.44 -0.85)		1.25(1.01 -1.54)		0.77(0.61 -0.98)	
**Histology**					/	/						
ADC											1	0.08
Non-ADC											1.33(0.97 -1.83)	
**ECOG**												
0-1	1	0.07	1	0.004*			1	0.035*	1	0.047*	1	<0.001*
≥2	0.72(0.51 -1.03)		0.57(0.39 -0.84)				1.54(1.03 -2.30)		1.28(1.00 -1.64)		0.60(0.46 -0.79)	
**Adrenal metastasis**												
Yes	1	0.018*	1	0.39								
No	0.63(0.43 -0.93)		0.84(0.56 -1.26)									

Abbreviations: ICI, Immune checkpoint inhibitors; AI, anti-angiogenesis inhibitors; NSCLC, non-small cell lung cancer; TX, treatment; ECOG PS, Eastern Cooperative Oncology Group performance status; ADC, adenocarcinoma; SQC, squamous cell carcinoma; HR, hazard ratio.

Supplementary Table S4. Survival of Patients in Different Groups Based on Demographic Factors

Characteristics	Docetaxel monotherapy				Docetaxel+Ramucirumab				Overall Docetaxel+/-Ramucirumab			
	PFS		OS		PFS		OS		PFS		OS	
	Median(95%Cl)	Log-rank P-value	Median(95%Cl)	Log-rank P-value	Median(95%Cl)	Log-rank P-value	Median(95%Cl)	Log-rank P-value	Median(95%Cl)	Log-rank P-value	Median(95%Cl)	Log-rank P-value
**Age**												
≥65	3.50(2.52-4.48)	0.59	7.30(5.58-9.02)	0.83	4.24(3.44-5.04)	0.97	8.60(5.99-11.21)	0.19	3.91(3.26-4.56)	0.79	7.86(6.40-9.33)	0.40
＜65	3.00(2.18-3.82)		7.90(5.39-10.41)		4.04(3.25-4.83)		9.30(6.81-11.79)		3.55(2.94-4.16)		8.67(6.93-10.42)	
**Gender**												
Male	3.25(2.61-3.89)	0.37	7.17(5.57-8.77)	0.68	3.83(2.99-4.68)	0.06	7.33(5.68-8.98)	0.005*	3.50(2.98-4.02)	0.04*	7.33(6.19-8.47)	0.02*
Female	3.30(2.44-4.16)		7.90(5.44-10.36)		4.83(4.05-5.61)		11.23(7.27-15.19)		4.01(3.05-4.97)		9.76(7.97-11.55)	
**Smoking history**												
Current/ Former	3.25(2.63-3.87)	0.71	7.30 (6.32-8.28)	0.09	4.18(3.67-4.69)	0.58	9.47(7.19-11.75)	0.95	3.85(3.34-4.36)	0.91	8.10(6.71-9.49)	0.24
Never	3.30(1.79-4.81)		11.80 (5.50-18.10)		3.50(2.01-4.99)		8.17(7.01-9.33)		3.43(2.18-4.68)		8.60(5.24-11.96)	
**Histology**												
ADC	3.20(2.57-3.83)	0.60	8.40(6.25-10.55)	0.07	4.27(3.72-4.82)	0.33	8.93(6.86-11.00)	0.11	3.85(3.29-4.41)	0.26	8.67(7.17-10.17)	0.02*
Non-ADC	3.55(2.37-4.73)		6.51(5.01-8.01)		3.45(1.60-5.30)		6.94(2.98-10.90)		3.45(2.77-4.13)		6.51(4.86-8.16)	
**ECOG**												
≥2	2.83(1.95-3.71)	0.08	6.64(4.80-8.48)	0.003*	3.45(2.25-4.65)	0.36	6.73(3.04-10.42)	0.02*	3.20(2.40-4.00)	0.05*	6.64(4.53-8.75)	<.001*
0-1	3.50(2.92-4.09)		8.40(6.15-10.65)		4.37(3.71-5.03)		9.30(6.99-11.61)		4.01(3.51-4.51)		8.93(7.39-10.47)	

**Line of treatment**			0.60		0.60		0.62		0.37		0.84		0.60
	≥3	3.40(2.68-4.12)		8.40(6.16-10.64)		4.40(3.88-4.93)		8.17(5.62-10.72)		3.90(3.33-4.47)		8.17(6.42-9.92)	
	2	3.00(2.20-3.80)		6.64(5.51-7.77)		3.83(2.85-4.81)		9.30(7.17-11.43)		3.43(2.72-4.14)		8.60(7.00-10.20)	
**Brain metastasis**			0.15		0.42		0.85		0.11		0.50		0.43
	Yes	2.87(2.08-3.66)		7.40(5.18-9.62)		4.17(3.59-4.75)		11.54(8.50-14.58)		3.50(2.71-4.29)		9.47(6.71-12.23)	
	No	3.68(2.97-4.39)		7.86(5.63-10.09)		4.17(3.18-5.16)		8.10(6.39-9.81)		3.90(3.30-4.50)		7.93(6.71-9.16)	
**Liver metastasis**			0.90		0.14		0.11		0.75		0.19		0.62
	Yes	3.00(0.32-5.68)		6.20(5.26-7.14)		4.14(2.79-5.49)		7.33(2.18-12.48)		3.00(1.76-4.24)		6.84(5.76-7.92)	
	No	3.30(2.70-3.90)		8.60(6.54-10.66)		4.27(3.79-4.75)		8.93(7.44-10.42)		3.80(3.30-4.30)		8.67(7.45-9.89)	
**Bone metastasis**			0.49		0.84		0.73		0.26		0.46		0.59
	Yes	3.00(2.38-3.62)		7.40(4.31-10.49)		4.17(3.67-4.67)		8.67(7.02-10.32)		3.70(3.70-4.28)		7.90(6.40-9.40)	
	No	3.72(2.72-4.72)		7.86(5.97-9.75)		4.17(2.95-5.39)		10.93(7.12-14.74)		3.90(3.30-4.50)		8.60(7.02-10.18)	
**Adrenal metastasis**			0.02*		0.36		0.38		0.40		0.35		0.88
	Yes	2.87(2.19-3.55)		6.64(1.79-11.49)		5.16(4.89-5.43)		10.93(2.30-19.56)		3.03(2.30-3.76)		8.63(5.26-12.00)	
	No	3.68(2.82-4.54)		7.86(5.75-9.97)		3.93(3.31-4.55)		8.67(7.14-10.20)		3.85(3.36-4.34)		8.17(6.98-9.37)	

Abbreviations: ADC, adenocarcinoma; ECOG, Eastern Cooperative Oncology Group performance status.

Supplementary Table S5. Justified HRs for Selected Molecular Markers in Patients with Docetaxel+/-Ramucirumab Treatment

Molecular alterations	Docetaxel monotherapy				Docetaxel+ramucirumab				Docetaxel+/- ramucirumab			
	PFS		OS		PFS		OS		PFS		OS	
	HR(95%Cl)	P value	HR(95%Cl)	P value	HR(95%Cl)	P value	HR(95%Cl)	P value	HR(95%Cl)	P value	HR(95%Cl)	P value
**TP53**												
TP53-mut	1	0.22	1	0.37	1	0.97	1	0.67	1	0.28	1	0.46
TP53-wt	0.81(0.58-1.13)		1.17(0.83-1.67)		1.01(0.74-1.37)		1.08(0.76-1.55)		1.13(0.91-1.41)		0.91(0.71-1.17)	
**KRAS**		0.94		0.94		0.27		0.80		0.38		0.86
KRAS-mut	1		1		1		1		1		1	
KRAS -wt	0.99(0.69-1.41)		1.01(0.69-1.49)		1.19(0.87-1.62)		1.05(0.73-1.50)		1.11(0.88-1.40)		1.02(0.79-1.32)	
**EGFR**		0.48		0.09		0.37		0.42		0.89		0.09
EGFR-mut	1		1		1		1		1		1	
EGFR--wt	0.88(0.61-1.2)		0.70(0.46-1.06)		0.84(0.58-1.23)		1.21(0.77-1.91)		0.98(0.77-1.28)		1.31(0.96-1.78)	
**STK11**												
STK11-mut	1	0.55	1	0.24	1	0.40	1	0.16	1	0.96	1	0.26
STK11-wt	1.81(0.69-2.02)		1.42(0.80-2.52)		1.26(0.74-2.12)		0.80(0.59-1.09)		1.01(0.69-1.47)		0.80(0.53-1.19)	
**ERBB2(HER2)**												
ERBB2-mut	1	0.07	1	0.63	1	0.35	1	0.66	1	0.09	1	0.74
ERBB2-wt	1.77(0.95-3.29)		1.17(0.61-2.25)		1.30(0.75-2.27)		1.15(0.61 -2.16)		0.70(0.47-1.06)		0.93(0.59-1.45)	
**MET**												
MET-mut	1	0.49	1	0.37	1	0.34	1	0.90	1	0.37	1	0.26
MET-wt	0.83(0.48-1.42)		0.71(0.39-1.26)		1.25(0.74-2.12)		0.89(0.50-1.60)		1.01(0.64-1.47)		0.80(0.53-1.19)	
**PIK3CA**												
PIK3CA-mut	1	0.06	1	0.37	1	0.26	1	0.42	1	0.91	1	0.19

PIK3CA-wt	1.31 (0.88-2.21)	1.37(0.69-2.72)	1.56(0.72-3.34)	0.71(0.31-1.63)	0.97(0.58-1.61)	1.42(0.84-2.39)

Abbreviations: TP53: tumor protein 53; KRAS: Kirsten rat sarcoma virus; EGFR: epidermal growth factor receptor; BRAF: The V-Raf Murine Sarcoma Viral Oncogene Homolog B; STK11: serine/threonine kinase 11; ERBB2(Receptor tyrosine-protein kinase erbB-2); MET: The mesenchymal epithelial transition factor receptor; PIK3CA: phosphatidylinositol-4,5-bisphosphate 3-kinase catalytic subunit alpha.

Supplementary Table S6. Comparison of Distribution of Significant Factors Between Docetaxel+/-Ramucirumab and Platinum-Taxane Groups

	Docetaxel+/-Ramucirumab	Taxane+Platinum	P value
**Gender**			0.75
Male	195	36	
Female	186	37	
**Histology**			0.09
ADC	316	55	
Non-ADC	65	18	
**ECOG**			0.12
≥2	87	18	
0-1	294	55	
**Adrenal metastasis**			0.21
Yes	64	8	
No	317	65	
**Brain metastasis**			
Yes	154	24	0.23
No	227	49	

Abbreviations: ADC adenocarcinoma; ECOG, Eastern Cooperative Oncology Group performance status.
