# Supplementary material for: Clinical Benefit from Docetaxel +/− Ramucirumab Is Not Associated with Mutation Status in Metastatic Non-Small-Cell Lung Cancer Patients Who Progressed on Platinum Doublets and Immunotherapy
Source: Cancers (Basel). 2024 Feb 26;16(5):935. doi: 10.3390/cancers16050935 (PMC10931294; doi:10.3390/cancers16050935)
Supplement: Supplementary file 1 [file cancers-16-00935-s001.zip › Supplement files/Supplement2.pdf]

Supplementary Figure S1

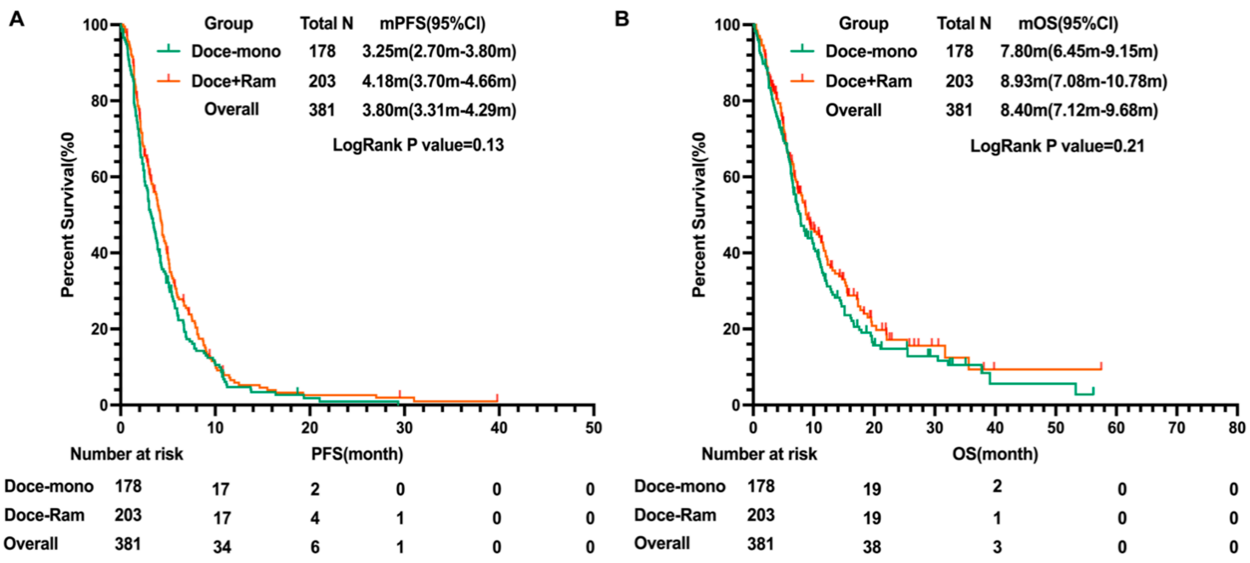

Supplementary Figure S2

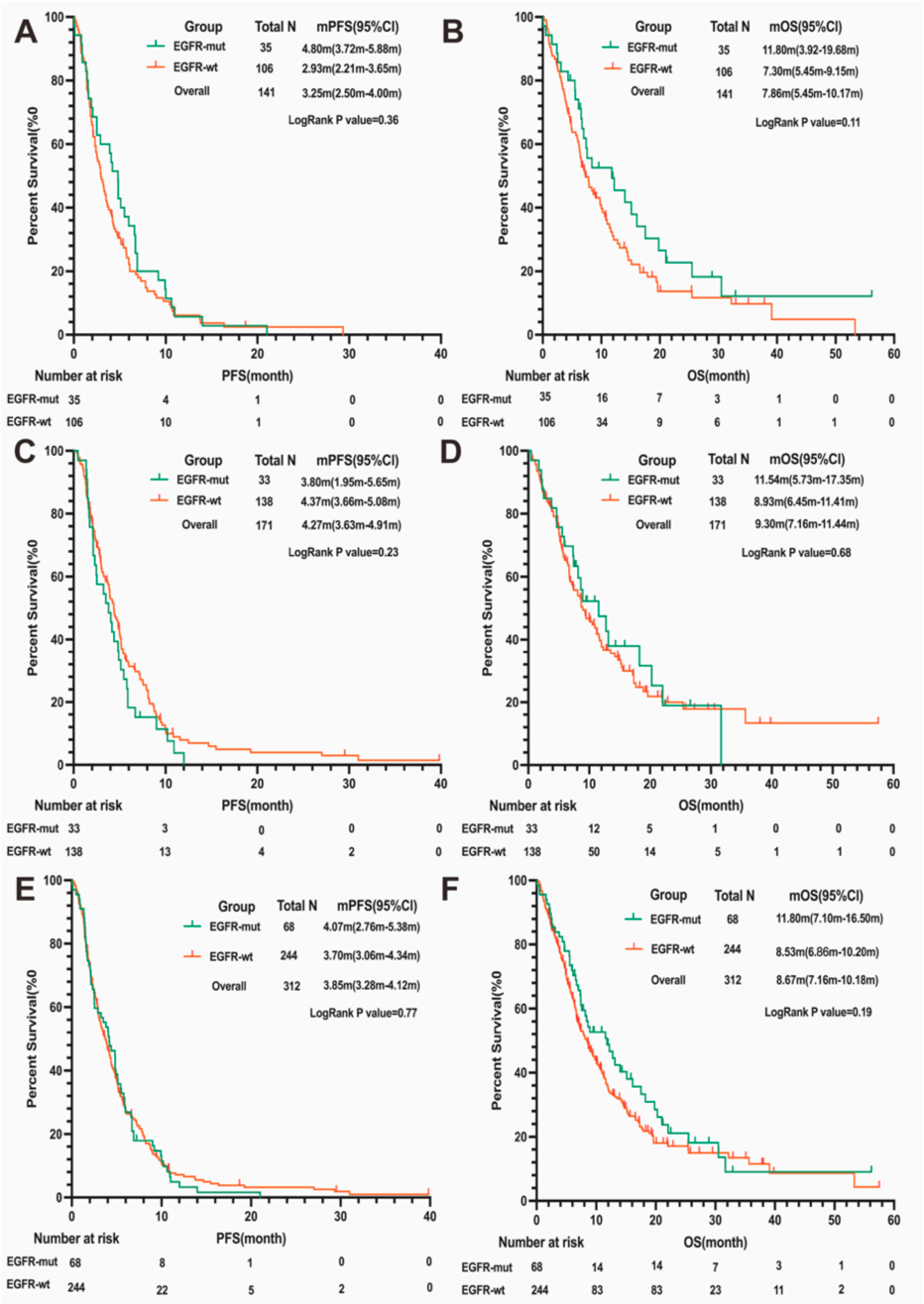

Supplementary Figure S3

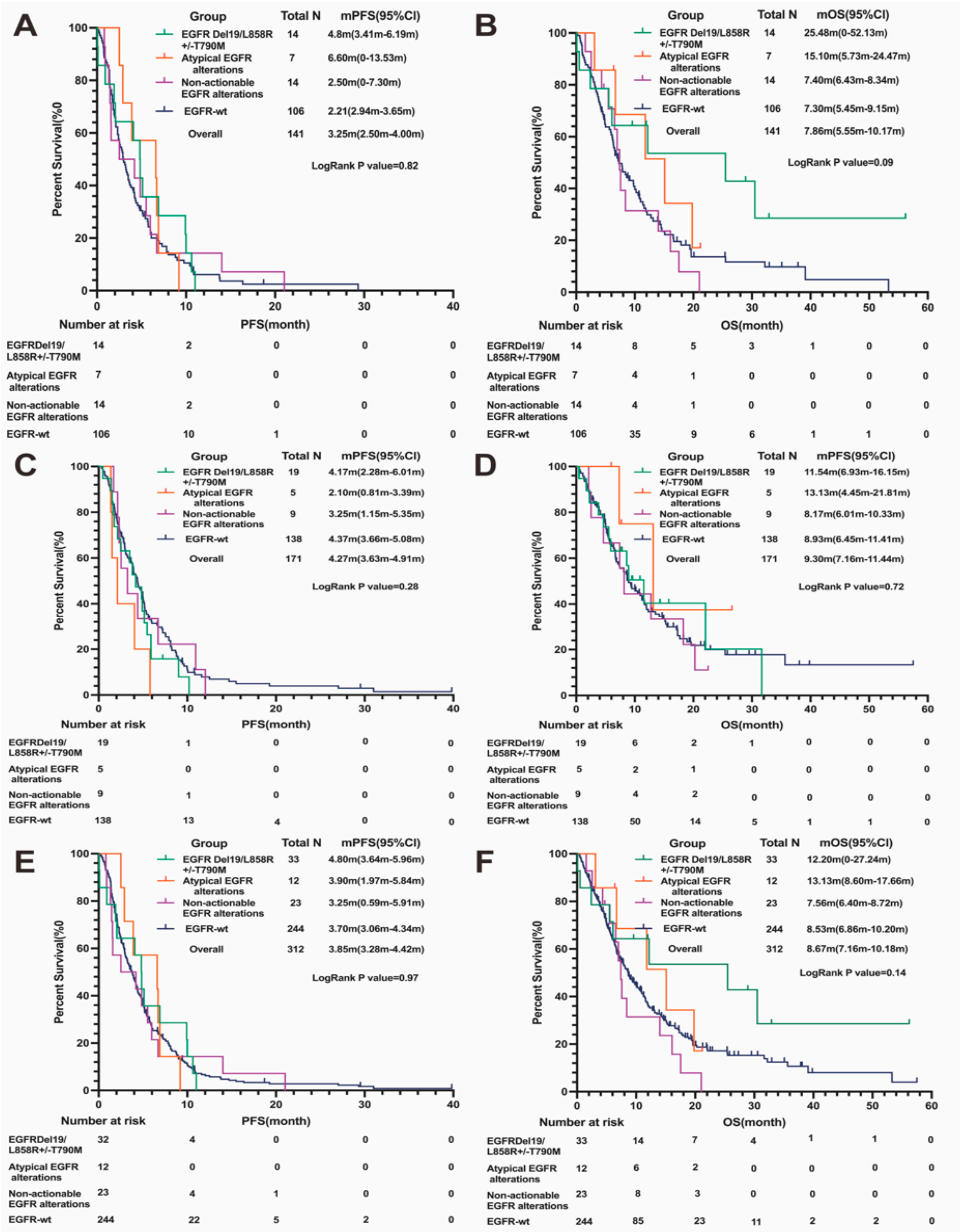

Supplementary Figure S4

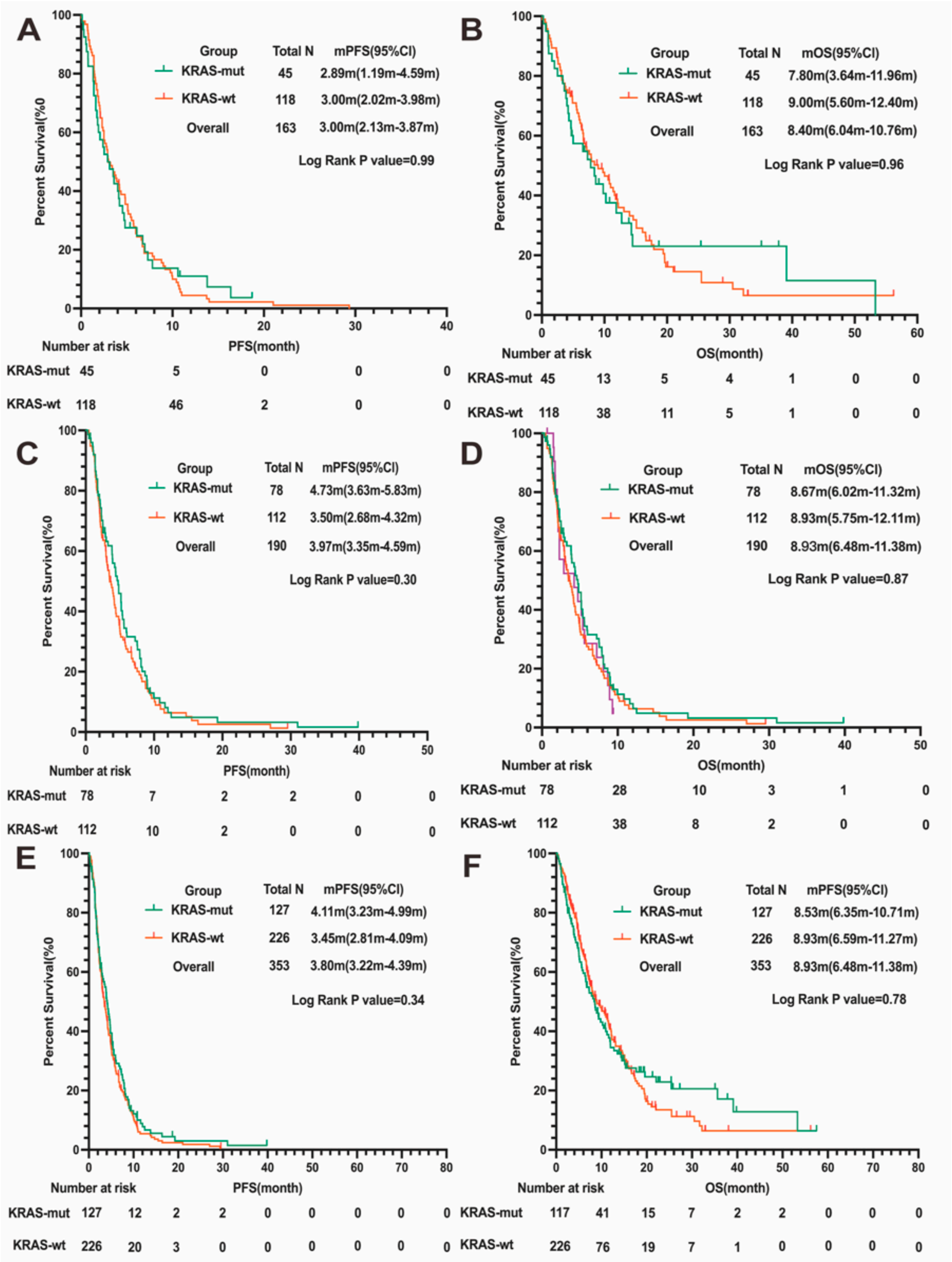

Supplementary Figure S5

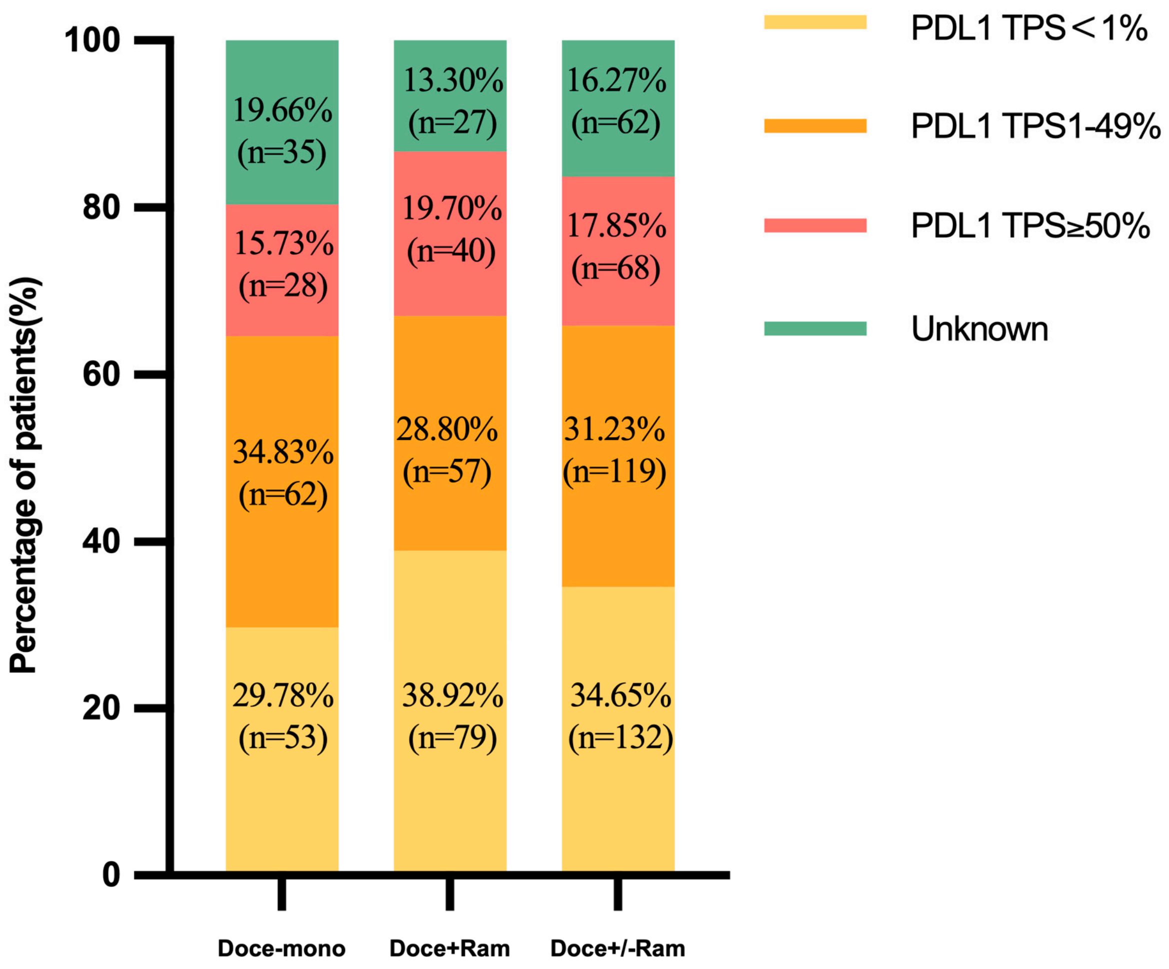

Supplementary Figure S6

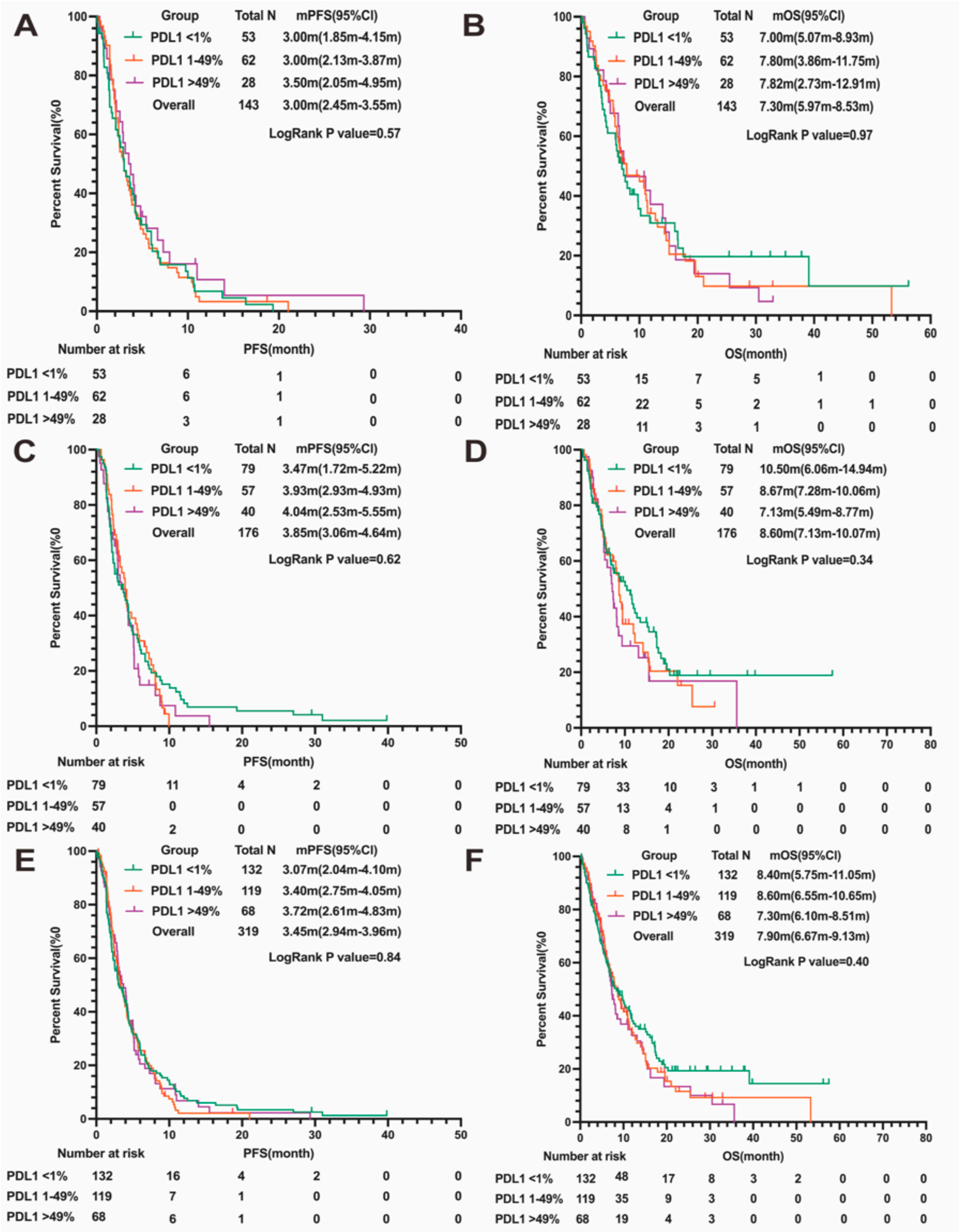

Supplementary Figure S7

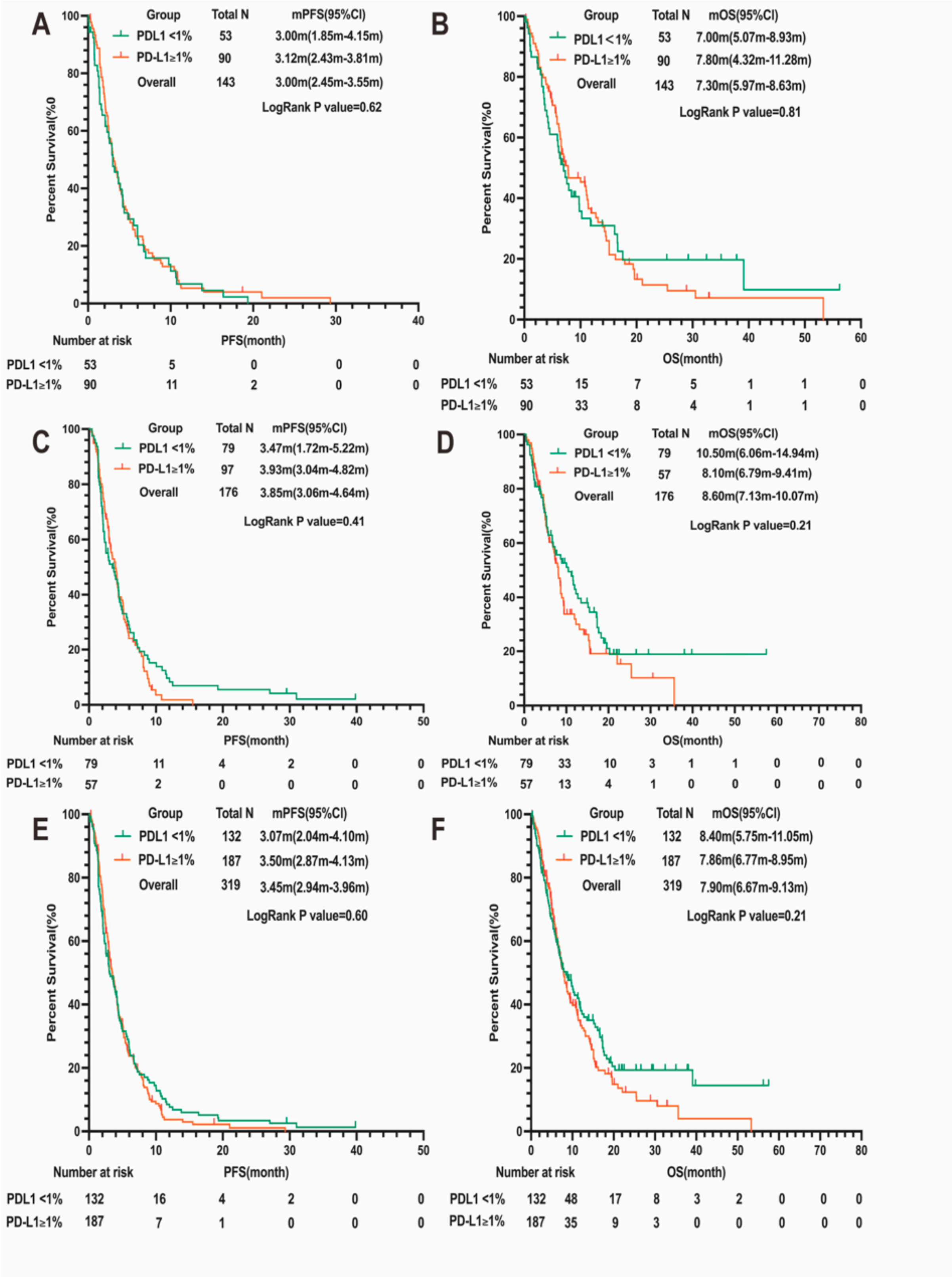

Supplementary Figure S8

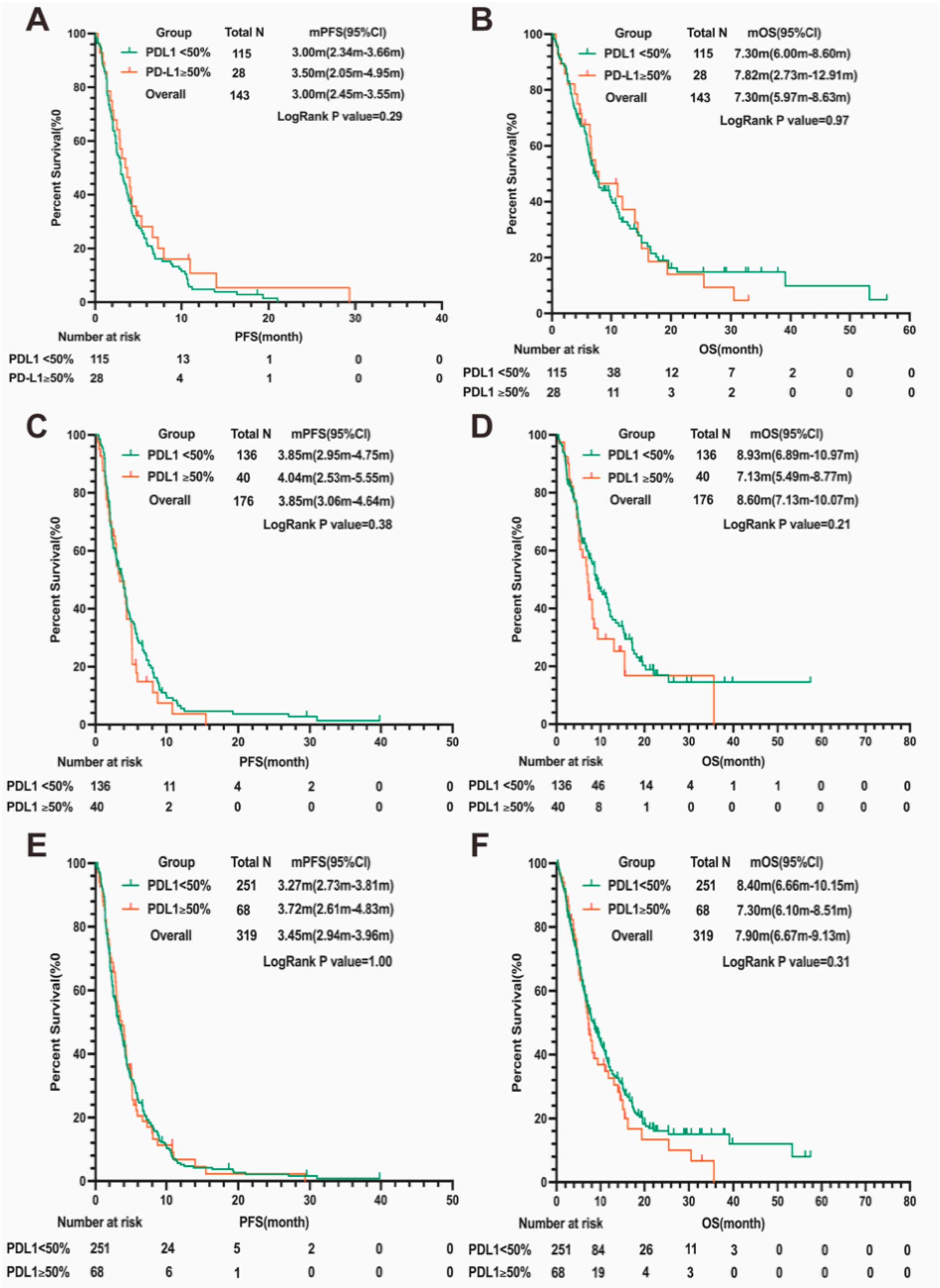

Supplementary Figure S9

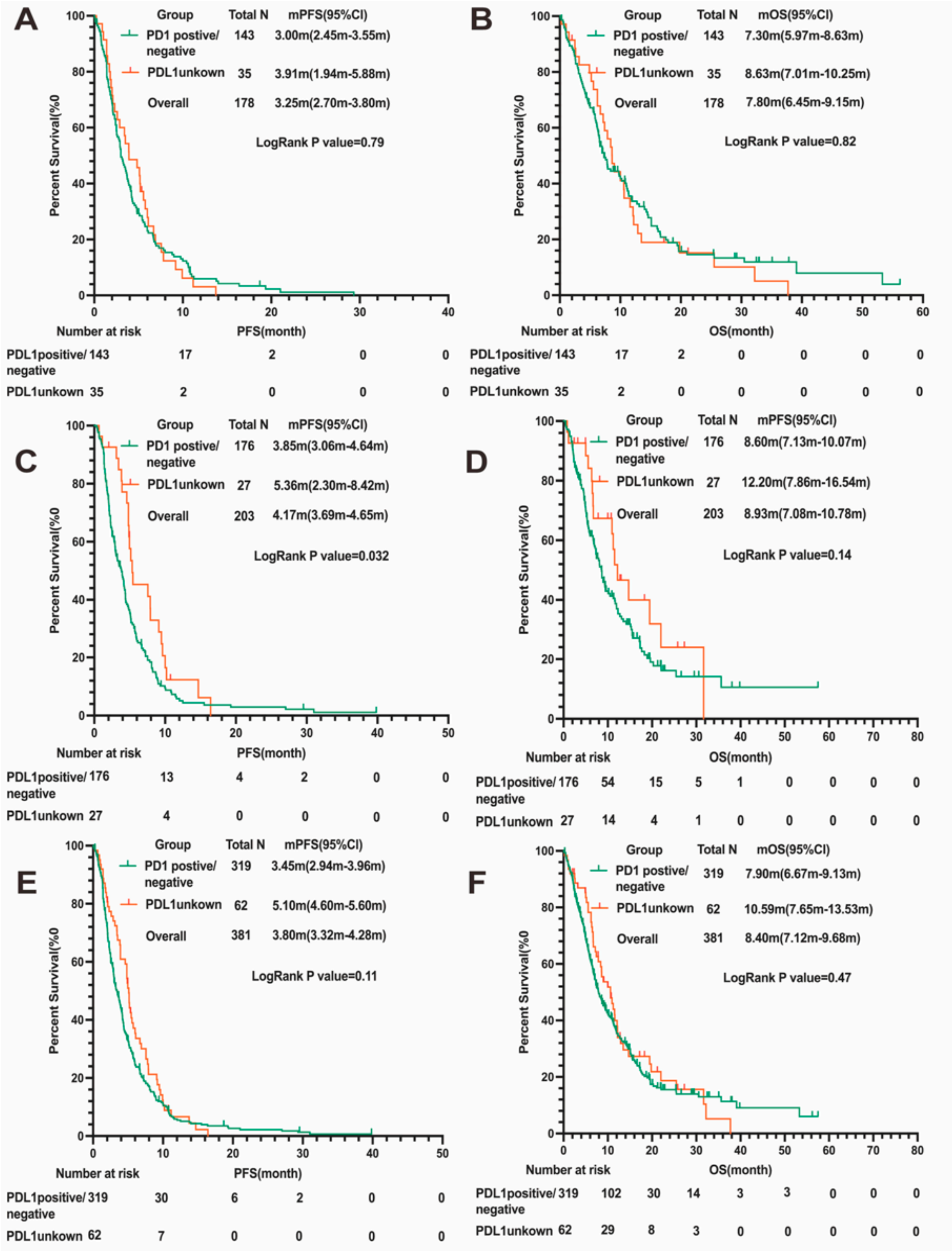

Figure Legends

Figure1. Comparison of PFS and OS in patients treated with docetaxel+/-ramucirumab versus platinum/taxane-based regimens. (A)PFS and **(B)**OS of patients treated with docetaxel+ramucirumab(N=381) versus platinum/taxane-based regimens(N=73); **(C)**PFS and **(D)** OS of patients treated with docetaxel+ramucirumab(N=20) versus platinum/taxane-based regimens(N=8) who developed disease progression within 2-4 months of initial platinum discontinuation following platinum doublet and ICI induction in the MDA cohort.

Abbreviations: PFS, progression-free survival; OS, overall survival; HR, hazard ratio; CI, confidence interval; Doce, docetaxel; Ram, ramucirumab; ICI: immune checkpoint inhibitor.

Supplementary Figure S1. Kaplan-Meier curve for PFS and OS in NSCLC patients with docetaxel+/-ramucirumab treatments(N=381). (A) PFS **(B)** OS of patients with docetaxel monotherapy and docetaxel+ramucirumab.

Abbreviations: PFS, progression-free survival; OS, overall survival; NSCLC, non-small cell lung cancer; HR, hazard ratio; CI, confidence interval; N, number; Doce, docetaxel; Ram, ramucirumab.

Supplementary Figure S2. Kaplan-Meier curve for PFS and OS in patients with adenocarcinoma with positive and negative EGFR alterations in each treatment group. (A) PFS and (**B)** OS of patients with docetaxel monotherapy(N=141); **(C)**PFS

and **(D)** OS of patients with docetaxel plus ramucirumab(N=171); **(E)** PFS and **(F)** OS of patients with treatment of docetaxel+/-ramucirumab(N=312).

Abbreviations: PFS, progression-free survival; OS, overall survival; NSCLC, non-small cell lung cancer; EGFR, epidermal growth factor receptor.

Supplementary Figure S3. Kaplan-Meier curve for PFS and OS in patients with adenocarcinoma with different subtype of EGFR alterations in each treatment group. (A)PFS and **(B)**OS of patients with docetaxel monotherapy(N=141); **(C)** PFS and **(D)**OS of patients with docetaxel plus ramucirumab(N=171); **(E)** PFS and **(F)** OS of patients with treatment of docetaxel+/-ramucirumab(N=312).

Abbreviations: PFS, progression-free survival; OS, overall survival; EGFR, epidermal growth factor receptor.

Supplementary Figure S4. Kaplan-Meier curve for PFS and OS in patients with adenocarcinoma with positive and negative KRAS alterations in each treatment group. (A) PFS and **(B)**OS of patients with docetaxel monotherapy(N=163); **(C)** PFS and **(D)**OS of patients with docetaxel plus ramucirumab(N=190); **(E)** PFS and **(F)** OS of patients with treatment of docetaxel+/-ramucirumab(N=353).

Abbreviations: PFS, progression-free survival; OS, overall survival; NSCLC, non-small cell lung cancer; HR, hazard ratio; CI, confidence interval; KRAS, Kirsten rat sarcoma virus.

Supplementary Figure S5. Percent of patients with different PD-L1 status (TPS＜1%，TPS1-49%，TPS＞50%, unknown) with docetaxel+/-ramucirumab treatment(N=381).

Abbreviations: Doce, docetaxel; Ram, ramucirumab; PD-L1, programmed death-ligand 1; TPS, tumor proportion score.

Supplementary Figure S6. Kaplan-Meier curve for PFS and OS in patients with different PD-L1 status (TPS score at 1% and 50% cutoff) in each treatment group. **(A)** PFS and **(B)**OS of patients with docetaxel monotherapy(N=143); **(C)** PFS and (D)OS of patients with docetaxel plus ramucirumab(N=176); **(E)** PFS and **(F)** OS of patients with treatment of docetaxel+/-ramucirumab(N=319).

Abbreviations: PFS, progression-free survival; OS, overall survival; NSCLC, non-small cell lung cancer; PD-L1, programmed death-ligand 1; TPS, tumor proportion score.

Supplementary Figure S7. Kaplan-Meier curve for PFS and OS in patients with different PD-L1 status (TPS at 1% cutoff) in each treatment group. **(A)** PFS and **(B)**OS of patients with docetaxel monotherapy(N=143); **(C)** PFS and **(D)**OS of patients with docetaxel plus ramucirumab(N=176); **(E)** PFS and **(F)** OS of patients with treatment of docetaxel+/-ramucirumab(N=319).

Abbreviations: PFS, progression-free survival; OS, overall survival; NSCLC, non-small cell lung cancer; PD-L1, programmed death-ligand 1; TPS, tumor proportion score.

Supplementary Figure S8. Kaplan-Meier curve for PFS and OS in patients with different PD-L1 status (TPS at 50% cutoff) in each treatment group. (A) PFS and **(B)**OS of patients with docetaxel monotherapy(N=143); **(C)** PFS and **(D)**OS of patients with docetaxel plus ramucirumab(N=176); **(E)** PFS and **(F)** OS of patients with treatment of docetaxel+/-ramucirumab(N=319).

Abbreviations: PFS, progression-free survival; OS, overall survival; NSCLC, non-small cell lung cancer; PD-L1, programmed death-ligand 1; TPS, tumor proportion score.

Supplementary Figure S9. Kaplan-Meier curve for PFS and OS in patients with different PD-L1 status (TPS known vs. unknown) in each treatment group. (A) PFS and **(B)**OS of patients with docetaxel monotherapy(N=178); **(C)** PFS and **(D)**OS of patients with docetaxel plus ramucirumab(N=203); **(E)** PFS and **(F)** OS of patients with treatment of docetaxel+/-ramucirumab(N=381).

Abbreviations: PFS, progression-free survival; OS, overall survival; NSCLC, non-small cell lung cancer; PD-L1, programmed death-ligand 1; TPS, tumor proportion score.
